# Osteogenic Sarcoma in an Adolescent With Cystic Fibrosis: Successful Treatment Despite Significant Obstacles

**DOI:** 10.3389/fped.2018.00245

**Published:** 2018-09-21

**Authors:** Thomas J. C. Ruffles, Ryan Black, Wayne Nicholls, Barbara Laing, Alan Isles

**Affiliations:** ^1^Department of Respiratory and Sleep Medicine, South Brisbane, QLD, Australia; ^2^Lady Cilento Children's Hospital and the Centre for Children's Health Research, South Brisbane, QLD, Australia; ^3^Department of Physiotherapy, South Brisbane, QLD, Australia; ^4^Oncology Services Group, Lady Cilento Children's Hospital, South Brisbane, QLD, Australia; ^5^Wesley Medical Imaging, Brisbane, QLD, Australia

**Keywords:** cystic fibrosis, osteogenic sarcoma, tibia, chemotherapy, non-tuberculous mycobacteria

## Abstract

**Introduction:** We describe the case of a 16-year old male with cystic fiborosis (CF) who presented with an osteosarcoma of his right distal tibia.

**Case Report:** Treatment consisted of neoadjuvant chemotherapy of cisplatin, doxorubicin and high dose methotrexate followed by distal tibial resection and free fibula flap reconstruction and consolidation chemotherapy. Treatment was complicated by a pulmonary exacerbation, where *Pseudomonas aeruginosa* (PsA) and *Staphylococcus aureus* were grown on sputum culture which was treated with a 2-week course of intravenous piptazobactam and tobramycin. *Mycobacterium intracellulare* and *Mycobacterium abscessus* were also cultured following commencement of chemotherapy and successfully treated with a 6-month course of oral azithromycin, ethambutol, and moxifloxacin along with a 1-month course of inhaled amikacin. Pulmonary function improved during his treatment from baseline FEV1 of 3.8 l (93.9%) to 4.15 l (102.3% predicted) whilst nutritional status remained stable.

**Discussion:** The combination of CF and osteosarcoma is rare with only one previous case reported (1). Our case is instructive as the patient faced the challenge of chronic PsA and the first reported culturing and successful treatment of non-tuberculous mycobacterium (NTM) during chemotherapy. Fatal outcomes have been reported previously for CF patients during immunosuppression (2). In concordance with our findings, a recent report noted an improvement in respiratory function in a child treated for leukemia (3). The anti-inflammatory nature of some chemotherapy agents could be responsible for the observed clinical improvement in CF with low dose methotrexate having been shown to increase FEV1 in adolescents with advanced CF (4). Whilst doxorubicin could improve pulmonary outcomes through increased total cellular CFTR protein expression and CFTR associated chloride secretion (5). It is hypothesized that the improved pulmonary function in patients with CF who require chemotherapy could be due to increased production of Multi-Drug Resistance Proteins (MDR) and Multi-Drug Resistant Associated Proteins (MRP) that may complement the depleted CFTR protein (6).

**Concluding Remarks:** We report the well-tolerated management of osteosarcoma in a patient with CF including the first reported identification and eradication of NTM during chemotherapy. The observed positive pulmonary outcome following chemotherapy highlights several potential cellular mechanisms that deserve to be explored.

## Introduction

The overall burden of cancer in patients with cystic fibrosis (CF) is low. However, multi-center, long-term cohort studies have shown some increased risk of malignancy in persons with CF (7). This risk increases after organ transplantation (8). We describe a 16-year old male with CF who presented with an osteosarcoma of his right distal tibia.

Our current case is instructive in that he underwent pre- and post-operative chemotherapy despite the long-term presence of *Pseudomonas aeruginosa* (PsA) in his sputum and culturing non-tuberculous mycobacteria (NTM) during his treatment.

## Case report

**Figure 1:**
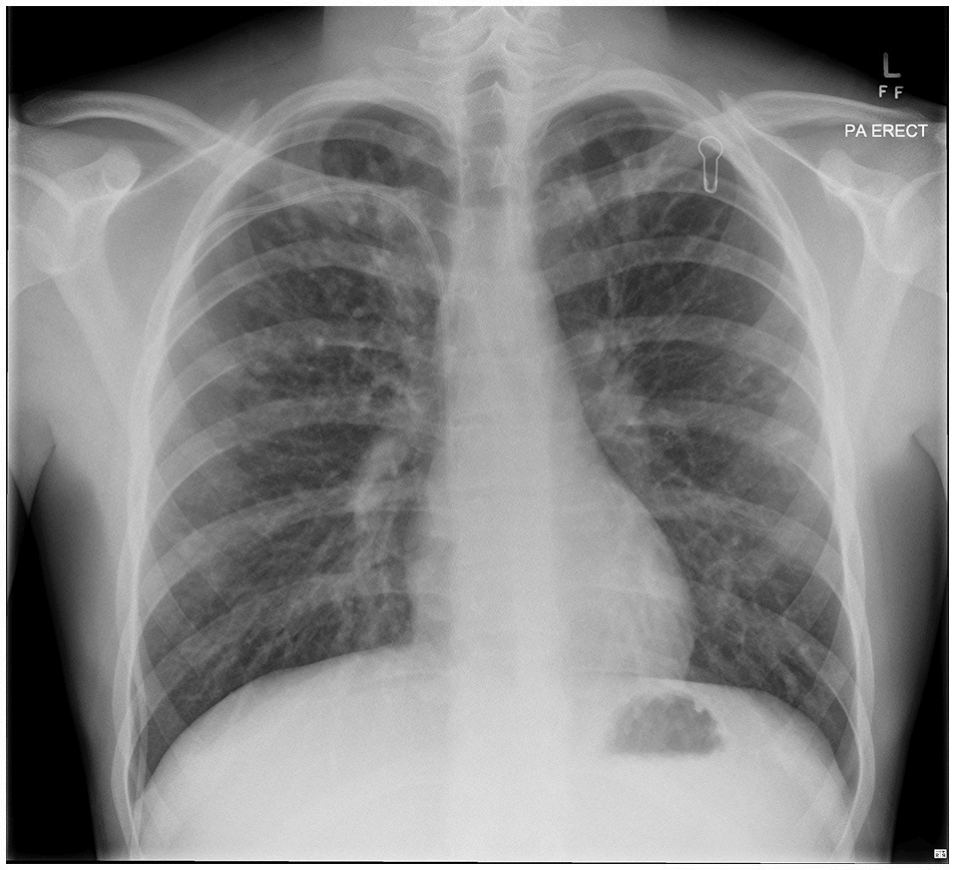
Chest x-ray, prior to commencing treatment.

**Figure 2:**
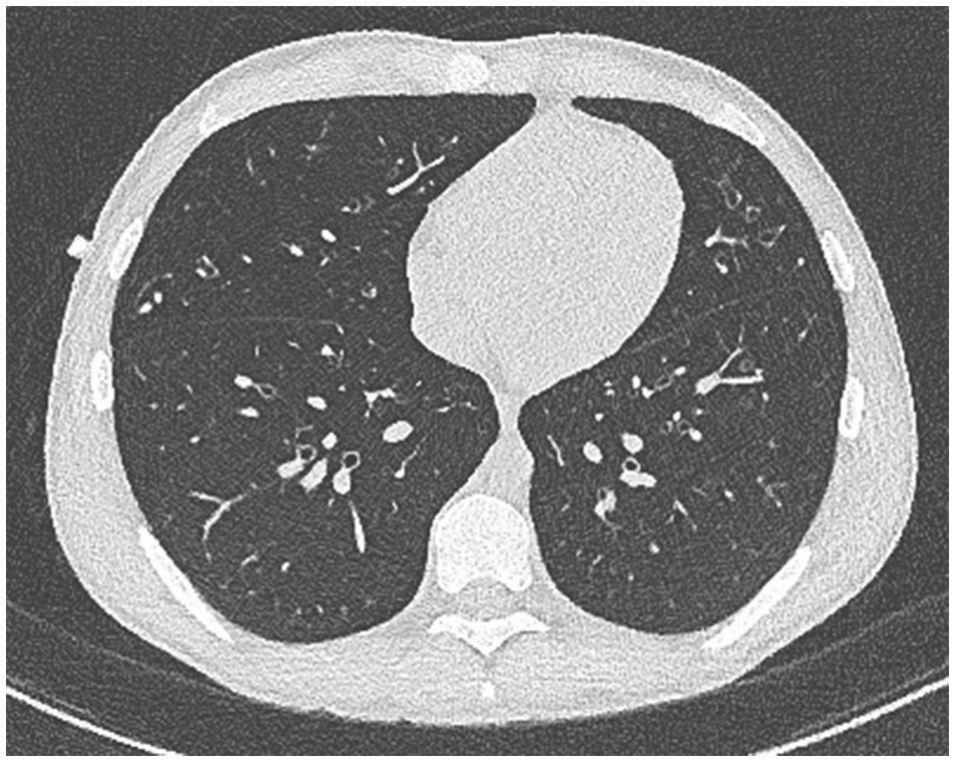
High resolution CT chest with contrast. Generalized bronchiectasis demonstrated.

Our patient with CF (homozygous for ΔF508 genotype) was diagnosed following newborn screening. He was from a rural Queensland family who lived some distance from tertiary CF services. Despite HRCT evidence of progressive bronchiectasis (see Figures 1, 2), growth and development was excellent (Weight and height 64th centile) with baseline FEV_1_ 3.8 l (93.9%). He had previously cultured *Staphylococcus aureus* (SA) and PsA (non-mucoid) and had one admission in the previous 12 months, requiring a pulmonary optimization with intravenous Piptazobactam and Tobramycin following a period of wet cough and decline in lung function.

**Figure 3:**
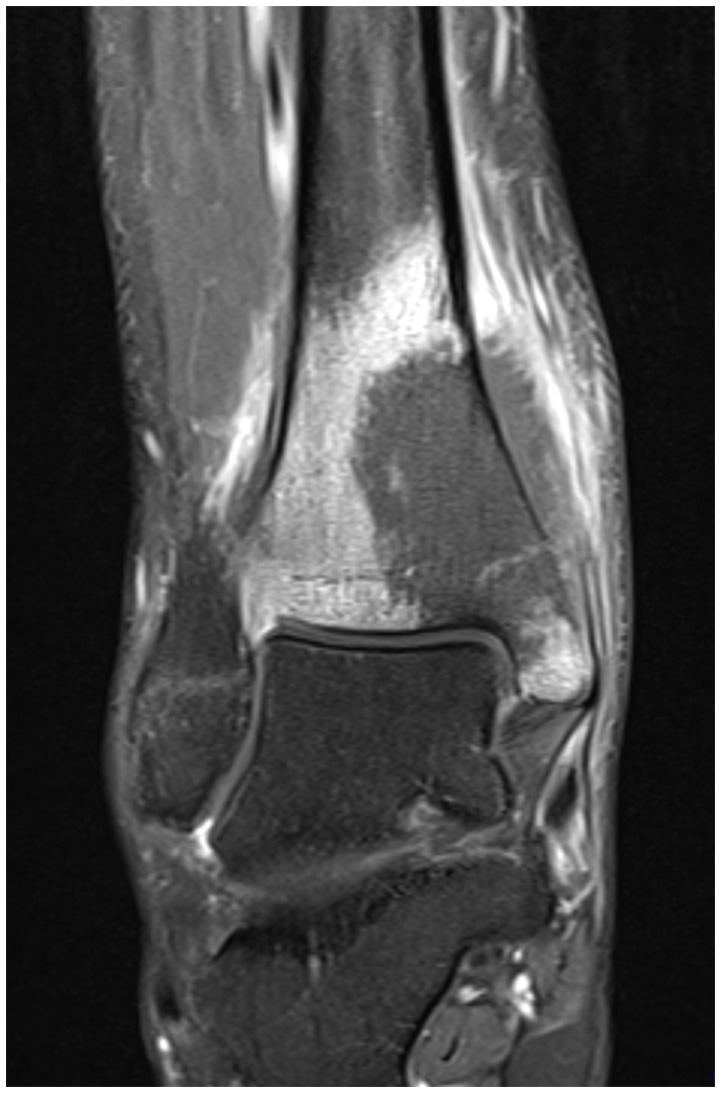
MRI, coronal section of right ankle, T1 weighted image.

He presented to his family doctor with a 3-month history of a progressively painful swollen right ankle. He was referred to an orthopedic specialist in a near-by provincial center where an MRI identified a suspected tumor in his distal tibia (see Figure 3). A biopsy confirmed the diagnosis to be a malignant fibrous histiocytoma type osteosarcoma. No systemic metastases were detected.

Treatment was initiated using protocol AOST0331 consisting of two 5-week cycles of neoadjuvant induction chemotherapy of cisplatin, doxorubicin and high dose methotrexate followed by surgery and consolidation therapy consisting of 4 monthly cycles. Our patient was supported with GSCF after each cycle to minimize duration of myelosuppression to ameliorate the risk of neutropenia and transfusion burden. In-view of impending immunosuppression he was commenced on prophylactic cotrimoxazole and voriconazole.

Two sputum cultures were sent immediately prior to starting chemotherapy, given the patient was asymptomatic he was not commenced on targeted anti-microbial whilst the culture results were awaited. Two days after initiation of chemotherapy he developed wet cough and was productive of green sputum with an associated significant reduction in FEV_1_ to 2.96 l (69% predicted). Sputum culture grew PsA (non-mucoid) and SA. He was started on twice daily hypertonic saline nebulisers and completed 2 weeks of treatment using intravenous Piptazobactam and Tobramycin.

Seven days after commencing chemotherapy repeat sputum was PCR positive for NTM with culture growing *Mycobacterium intracellulare* and *Mycobacterium abscessus* subspecies *abscessus*. Given the risk of disseminated disease in patients who are immunosuppressed he was commenced on a 6-month course of oral azithromycin, ethambutol, and moxifloxacin along with a 1-month course of inhaled amikacin.

**Figure 4:**
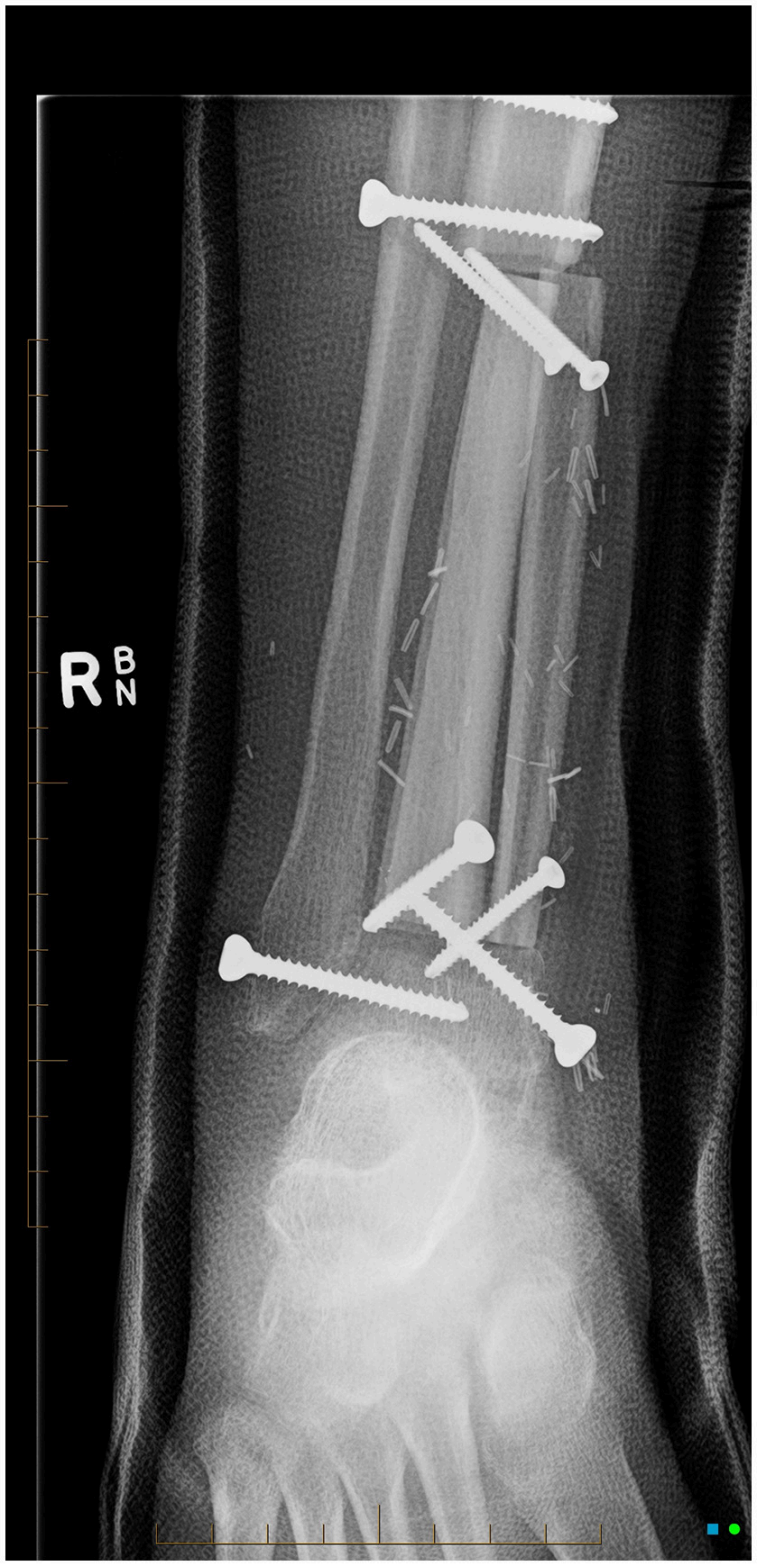
AP x-ray of right lower leg, cannulated screws transfix the fibular bone graft to the distal right tibial diaphysis.

**Figure 5:**
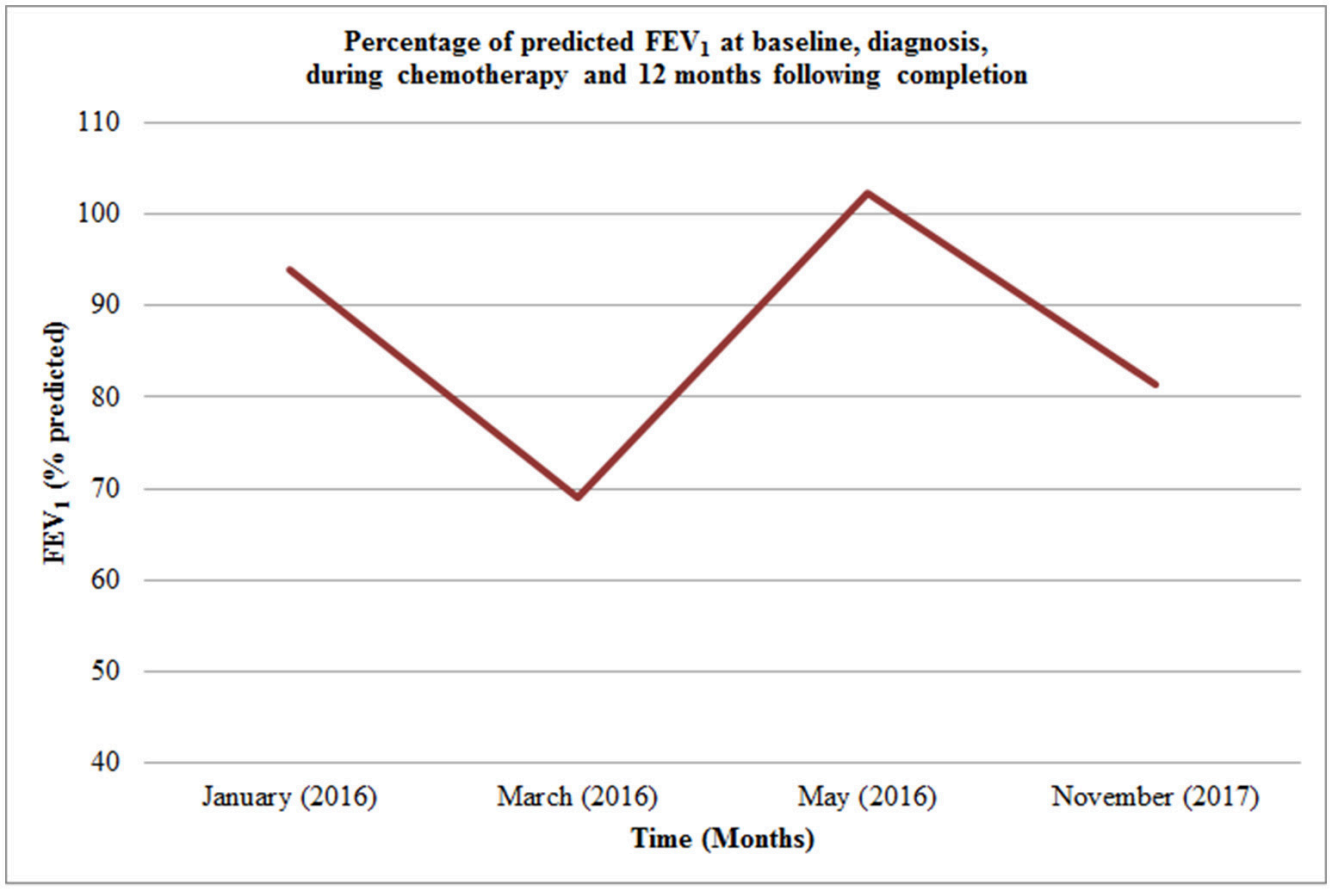
Percentage of predicted FEV_1_ at baseline, diagnosis, during chemotherapy and 12 months following completion.

Four months after diagnosis he underwent distal tibial resection and free fibula flap reconstruction (see Figure 4) which he recovered well from, requiring repeat bone graft to the right tibia 6 months later. Post-surgery consolidation chemotherapy consisted of cisplatin and doxorubicin, which was well-tolerated. Pulmonary function improved during his treatment from baseline (see Figure 5) with FEV_1_ 4.15 l (102.3% predicted) whilst nutritional status remained stable with weight 64.1 kg (62nd centile).

Twelve months post completion of consolidation chemotherapy and NTM eradication our patient is physically active and is in the process of begin transitioned to adult care. His lung function was reduced with FEV_1_ 3.49 l (81.3% predicted) in the context of a short duration wet cough that was treated with 2 weeks of Tobramycin nebulisers and oral ciprofloxacin. He has normal hepatic function, renal function, audiometry, and echocardiography. There has been no further mycobacterium obtained on repeated sputum culture.

His treatment was planned conjointly by the oncology and respiratory teams. There was an obvious concern that immunosuppression from chemotherapy in the presence of PsA and NTM might lead to significant pulmonary complications. What might have otherwise been complex decisions were simplified by the knowledge that without best-practice treatment for his sarcoma, eventual death was the expected outcome for our patient. The role of the Respiratory team then became that of supporting the oncology team and monitoring our patient through the planned cycle of treatment and surgery.

## Discussion

As people with CF live longer in addition to age-related CF complications, new life risks such as cancer arise. Two large US cohort studies have reported that while the risk of cancer in CF is low, there is an increased risk of gastrointestinal cancer as well as leukemia and testicular cancer (7, 8).

There has only been one previous report by Okuda et al. (1) of osteogenic sarcoma in a patient with CF; a 5-year old with a high-grade sarcoma of the humerus. These authors reported that the coincidence of CF and osteosarcoma was calculated to be as low as 1.76 x 10^−9^. In the German case report, the child's airway was colonized at various times before the cancer diagnosis with SA, *Serratia marcesens* as well as other common respiratory organisms. He received prophylactic intravenous cefuroxime during neutropenic phases. Two febrile neutropenic episodes were treated with other antibiotics according to standard protocols. The authors concluded that antineoplastic treatment was manageable without exacerbation of CF lung disease.

Our case is instructive as the patient faced different challenges with the long-term presence of PsA in his sputum and with culturing NTM during his treatment.

Previously fatal outcomes have been reported for patients with CF due to haemorrhagic bronchopneumonia secondary to PsA infection during immunosuppression (2). In concordance with our findings, a more recent case report noted an improvement in respiratory function in a 7-year-old boy treated for acute myeloid leukemia (AML) (3).

It is suggested that the anti-inflammatory nature of some of the chemotherapy agents could be responsible for the observed clinical improvement in CF. Our patient was treated with neoadjuvant methotrexate, cisplatin, and doxorubicin with the latter two agents being used in the consolidation phase. Ballman et al reported a median 9% improvement in FEV_1_ in five children and adolescents with advanced CF who were treated with low dose methotrexate for 12 months (4).

Other chemotherapy agents may potentially improve pulmonary function in CF by effecting cellular protein production with doxorubicin, that was used in the chemotherapy regimen for our patient, having been found to increase total cellular CFTR protein expression and CFTR associated chloride secretion in epithelial cells (9).

It has been postulated that improved pulmonary function in patients with CF who require chemotherapy may be due to increased production of Multi-Drug Resistance Proteins (MDR) and Multi-Drug Resistant Associated Proteins (MRP) that may complement the depleted CFTR protein (5). MDR and MRP are in the same class of membrane spanning transport proteins as CFTR and exhibit significant sequence homology. It is hypothesized that MRP and CFTR may both work by stimulating cellular removal of inflammatory glutathione adducts (5). Authors have recommended the collection of pre- and post-treatment sweat tests, nasal potential difference measurements and nasal brushings for quantification of MDR P glycoprotein mRNA to help further evaluate whether this mechanism could explain the observed improvement in respiratory status in some patients with CF treated with chemotherapy (3).

Given the multiple variables involved in our patient's management it is only possible to speculate what may have resulted in the observed improvement in his lung function. It is likely that the NTM eradication protocol consisting of oral azithromycin, ethambutol, and moxifloxacin along with inhaled amikacin, initiated 1 week after commencing chemotherapy, may have also contributed by reducing overall bacterial carriage. Azithromycin was shown in a recent Cochrane review to result in 3.97% increase in FEV_1_ and a reduction in exacerbation frequency (6). Whilst a phase two study of inhaled amikacin in adults showed a statistically significant improvement in FEV_1_ and a reduction in PsA sputum culture (10).

There are potential pulmonary complications to osteosarcoma treatment, most notably with methotrexate whilst doxorubicin and cisplatin are not known to be associated with respiratory specific side effects. Methotrexate related pulmonary toxicity observed in 0.5% of patients per year who take low dose methotrexate for inflammatory conditions, however it is less commonly associated with the high dose methotrexate therapy used in osteosarcoma treatment (11). Methotraxate induced pneumonitis is thought to be caused by an idiosyncratic hypersensitivity reaction and characteristically presents with progressive cough and dyspnoea with or without fever (12).

CT characteristically shows ground glass opacities with or without focal consolidation, evolving restrictive lung disease may help aide the diagnosis whilst an elevated CD4/CD8 ratio on bronchoalveolar lavage has also been shown (13). Non-specific Interstitial Pneumonia (NSIP) is the most common biopsy finding (14). Methotrexate cessation will often result in clinical resolution whilst corticosteroids are often used to treat the hypersensitivity pneumonitis (13). Thoracic metastatic osteosarcoma is commonly treated with radiation therapy with potential complications which can present within 6 months of treatment as radiation pneumonitis or later as radiation induced fibrosis (15).

The American Thoracic Society guideline for NTM diagnosis requires at least two separate positive sputum cultures or one positive culture from bronchoalveolar lavage to support a microbiological diagnosis (16). The decision to commence immediate treatment was made on the basis of the risk of disseminated disease that has been reported during anti-neoplastic chemotherapy (17).

Our patient was commenced on a 6-month treatment of oral azithromycin, ethambutol, and moxifloxacin along with a 1-month course of inhaled amikacin protocol having had a single sputum culture positive for *Mycobacterium intracellulare* and *Mycobacterium abscessus* soon after commencing chemotherapy. This treatment regimen is in line with the US CF Foundation and the European CF Society guidelines for treatment of the slow growing *Mycobacterium avium* complex (MAC) organisms such as *Mycobacterium intracellulare* which is more commonly associated with disseminated disease in immunocompromised hosts compared to rapid growing *Mycobacterium abscessus* complex (MABSC) (17). The primary aim of the treatment was to prevent disseminated disease and it covered the time period that our patient was immunocompromised by chemotherapy as opposed to the full 12 months treatment advised following culture conversion (18).

## Concluding remarks

We report the well-tolerated medical and surgical management of localized osteosarcoma in a patient with cystic fibrosis including the first reported identification and eradication of NTM during chemotherapy treatment. The observed positive pulmonary outcome following chemotherapy highlights several potential cellular mechanisms that deserve to be explored for possible therapeutic approaches for children with CF.

## Ethics statement

Written informed consent was obtained from the patient and the parents of the patient for the publication of this case report.

## Author contributions

AI and TR coordinated the writing group. AI and TR performed the literature review. All authors critically reviewed the manuscript, read, and approved the final version.

### Conflict of interest statement

The authors declare that the research was conducted in the absence of any commercial or financial relationships that could be construed as a potential conflict of interest.

## Abbreviations

CF: cystic fibrosis
NTM: non-tuberculous mycobacterium
PsA: *Pseudomonas aeruginosa*
SA: *Staphylococcus aureus*
ΔF508: Delta-F508
HRCT
MRI: Magnetic Resonance Imaging
FEV_1_: Forced Expressed Volume in 1 s
GCSF: Granulocyte Colony-Stimulating Factor
PCR: Polymerase Chain Reaction
MDR: Multi-Drug Resistance Proteins
MDP: Multi-Drug Resistant Associated Proteins
CFTR: Cystic Fibrosis Transmembrane Regulator
MAC: *Mycobacterium avium* complex
MABSC: *Mycobacterium abscessus* complex
NSIP: Non-specific Interstitial Pneumonia.
